# Slitrk2 controls excitatory synapse development via PDZ-mediated protein interactions

**DOI:** 10.1038/s41598-019-53519-1

**Published:** 2019-11-19

**Authors:** Kyung Ah Han, Jinhu Kim, Hyeonho Kim, Dongwook Kim, Dongseok Lim, Jaewon Ko, Ji Won Um

**Affiliations:** Department of Brain and Cognitive Sciences, Daegu Gyeongbuk Institute of Science and Technology (DGIST), 333 Techno Jungangdae-Ro, Hyeonpoong-eup, Dalseong-gun, Daegu 42988 Korea

**Keywords:** Molecular neuroscience, Development of the nervous system

## Abstract

Members of the Slitrk (Slit- and Trk-like protein) family of synaptic cell-adhesion molecules control excitatory and inhibitory synapse development through isoform-dependent extracellular interactions with leukocyte common antigen-related receptor protein tyrosine phosphatases (LAR-RPTPs). However, how Slitrks participate in activation of intracellular signaling pathways in postsynaptic neurons remains largely unknown. Here we report that, among the six members of the Slitrk family, only Slitrk2 directly interacts with the PDZ domain-containing excitatory scaffolds, PSD-95 and Shank3. The interaction of Slitrk2 with PDZ proteins is mediated by the cytoplasmic COOH-terminal PDZ domain-binding motif (Ile-Ser-Glu-Leu), which is not found in other Slitrks. Mapping analyses further revealed that a single PDZ domain of Shank3 is responsible for binding to Slitrk2. Slitrk2 forms *in vivo* complexes with membrane-associated guanylate kinase (MAGUK) family proteins in addition to PSD-95 and Shank3. Intriguingly, in addition to its role in synaptic targeting in cultured hippocampal neurons, the PDZ domain-binding motif of Slitrk2 is required for Slitrk2 promotion of excitatory synapse formation, transmission, and spine development in the CA1 hippocampal region. Collectively, our data suggest a new molecular mechanism for conferring isoform-specific regulatory actions of the Slitrk family in orchestrating intracellular signal transduction pathways in postsynaptic neurons.

## Introduction

Synapses are asymmetric intercellular junctions that permit transmission of signals from presynaptic to postsynaptic neurons. The transmission of synaptic information is coordinated by distinct multiprotein complexes that are compartmentalized at the presynaptic active zone, synaptic cleft, and postsynaptic density^1^. In particular, synaptic cell-adhesion molecules have been recognized as key components that bidirectionally organize the transfer and processing of synaptic information^2^. These proteins are thought to not only mediate the physical alignment of pre- and postsynaptic neurons, but also to orchestrate multiple *trans*-cellular signaling cascades in both pre- and postsynaptic neurons, lending specific properties to synapse types. Although a variety of synaptic cell-adhesion molecules have been recently demonstrated to organize ‘extracellular’ synaptic adhesion pathways, how diverse extracellular signals are directionally transduced, and whether different synaptic adhesion pathways can activate distinct ‘intracellular’ signaling pathways, remains largely unknown. Protein phosphorylation is one of the crucial mechanisms in the regulation of neural function, and a subset of synaptic cell-adhesion molecules directly regulate aspects of this process, particularly tyrosine phosphorylation and dephosphorylation, mediated by receptor tyrosine kinases and receptor tyrosine phosphatases, respectively (reviewed in^3^).

Slit- and Trk-like (Slitrk) family proteins, composed of six members (Slitrk1–6), are highly expressed in the central nervous system of vertebrates and are postsynaptically localized, controlling both excitatory and inhibitory synapse development^4–7^. All six members of the Slitrk family are structurally similar, particularly in extracellular sequences, which mediate interactions with three members of the leukocyte common antigen-related receptor tyrosine phosphatase (LAR-RPTP) family–PTPσ, PTPδ, and LAR–through the first cluster of leucine-rich repeat (LRR) domains^5,8,9^. Intriguingly, Slitrk family proteins control formation of distinct synapse-types in an isoform-dependent manner^10^. Although distinct extracellular interactions of Slitrks with specific LAR-RPTP family members likely shapes the establishment of specific adhesion pathways at distinct synapse types, it is plausible that unique intracellular signaling pathways underlying individual Slitrk members are also involved, in keeping with sequence divergence in intracellular sequences of Slitrk family members^11^.

Here, we report that Slitrk2, an excitatory synapse-specific Slitrk, directly binds to the PDZ (PSD-95/Dlg/ZO-1)-containing excitatory scaffolds, PSD-95 and Shank3. To explore the functions of Slitrk2-PDZ interactions, we performed various experiments in both cultured hippocampal neurons and hippocampal CA1 pyramidal neurons *in vivo*, revealing that PDZ proteins positively regulate excitatory synaptic targeting of Slitrk2, as well as Slitrk2-mediated excitatory (but not inhibitory) synapse formation and transmission.

## Materials and Methods

### Animals

All animal experiments were approved by the Institutional Animal Care and Use Committee (IACUC) of the Daegu Gyeongbuk Institute of Science and Technology (DGIST), and have been performed accordingly. All methods were performed in accordance with the relevant guidelines and protocols (DGIST-IACUC-17122104-01). Mice (C57BL/6N) for viral injection and pregnant rats for primary cultures were purchased from Daehan Biolink.

### Expression vectors

pRK5-FLAG-tagged rPSD-95 was generated by PCR amplification of rat PSD-95 (amino acids [aa] 1–724), followed by digestion with *Mlu*I and *Not*I, and cloning into the pRK5-FLAG vector, respectively. pRK5-FLAG-rPSD-95 ΔPDZ1 + 2 was generated by PCR amplification of rat PSD-95 (aa 303–724), followed by digestion with *Mlu*I and *Not*I, and cloning into the pRK5-FLAG vector. pRK5-FLAG-rPSD-95 ΔPDZ3 was generated by PCR amplification of rat PSD-95 fragments (aa 1–302 and aa 404–724), followed by digestion with *MluI* and *NotI* for the first fragment (aa 1–302) and *Not*I for the second fragment (aa 404–724), and subsequent cloning into the pRK5-FLAG vector. pRK5-FLAG-rPSD-95 ΔSH3 + GK was generated by PCR amplification of rat PSD-95 (aa 1–403), followed by digestion with *Mlu*I and *Not*I, and cloning into the pRK5-FLAG vector. pEGFP-C1-Shank3a ANK, pEGFP-C1-Shank3a SH3, pEGFP-C1-Shank3a PDZ, pEGFP-C1-Shank3a PR, and pEGFP-C1-Shank3a SAM constructs were generated by PCR amplification of the following Shank3 fragments: ANK, aa 104–347; SH3, aa 462–531; PDZ, aa 556–663; PR, aa 662–1673; and SAM, aa 1654–1728. These fragments were then digested with *EcoR*I and *BamH*I, and cloned into the pEGFP-C1 vector (Clontech). pDisplay-HA-hSlitrk2 ΔPBM was generated by PCR amplification of human Slitrk2 (aa 22–841), followed by digestion with *Xma*I and *Sac*II, and cloning into the pDisplay vector (Invitrogen). pAAV-Slitrk2 WT and pAAV-Slitrk2 ΔPBM were constructed by PCR amplification using pDisplay-hSlitrk2 WT and pDisplay-hSlitrk2 ΔPBM as templates, respectively, and subsequent subcloning into the pAAV2-T2A-EGFP vector at *Xba*I and *BamH*I sites. The following constructs were previously described: pDisplay-HA-hSlitrk1-5^10,12^; pEGFP-C1-mShank3 variants^13^; GW1-PSD-95^14^; L-315 and L-315 sh-Slitrk2^10^.

### Antibodies

His-tagged human GAD65 (aa 353–585) and GST-tagged rat PSD-95 (aa 1–724) were produced in *Escherichia coli* BL21 strain and purified by affinity chromatography, using a 400 mM imidazole solution (Affymetrix) or 10 mM L-Glutathione reduced (Sigma-Aldrich) to elute bound protein. Following immunization of a guinea pig with this immunogen, the GAD65-specific antibody (JK158) was affinity-purified using a Sulfolink column (Pierce) on which the same proteins were immobilized. A rat VGLUT1 peptide (GAETLELSADGRPVTTHTRDPPV) was synthesized and used for immunization in rabbits. VGLUT1-specific antibodies (JK111) produced from immunized rabbits were affinity-purified using a Sulfolink column (Pierce) on which the same peptides were immobilized. The following antibodies were obtained commercially: mouse monoclonal anti-HA (clone HA-7; Covance); mouse monoclonal anti-FLAG M2 (clone M2; Sigma-Aldrich); rabbit polyclonal anti-FLAG (Sigma-Aldrich); goat polyclonal anti-EGFP (Rockland Immunochemicals); guinea pig polyclonal anti-VGLUT1 (Millipore); rabbit polyclonal anti-VGLUT1 (Synaptic Systems); mouse monoclonal anti-PSD-95 (clone K28/43; NeuroMab); mouse monoclonal anti-β-actin (clone C4; Santa Cruz Biotechnology); rabbit polyclonal anti-Slitrk2 (ProSci Incorporated); and mouse monoclonal anti-CASK (clone K56A/50; NeuroMab). The following antibodies have been previously described: anti-PSD-95 [JK016]^15^, anti-S-SCAM [1146]^14^, anti-pan-Shank [1172]^16^, anti-PSD-93 [1634]^14^, and anti-SAP102 [1445]^14^.

### Coimmunoprecipitation assays

Brains (~2 g) from postnatal day 42 (P42) rats were homogenized in 10 ml ice-cold homogenization buffer consisting of 320 mM sucrose, 5 mM HEPES-NaOH (pH 7.5), 1 mM EDTA, 0.2 mM PMSF, 1 μg/ml aprotinin, 1 μg/ml leupeptin, 1 μg/ml pepstatin, and 1 mM Na_3_VO_4_. The homogenized tissue was centrifuged at 2000 × g for 15 min, after which the supernatant was centrifuged at 100,000 × g for 1 h. The pellets were homogenized in buffer consisting of 20 mM HEPES-NaOH (pH 7.5), 0.15 M NaCl, 2 mM CaCl_2_, 2 mM MgCl_2_, 0.2 mM PMSF, 1 μg/ml aprotinin, 1 μg/ml leupeptin, 1 μg/ml pepstatin, and 1 mM Na_3_VO_4_. Triton X-100 was added to a final concentration of 1% (w/v) and dissolved with constant stirring at 4 °C for 1 h. Supernatants obtained after centrifugation at 100,000 × g for 1 h were incubated with anti-Slitrk2 antibody overnight at 4 °C, followed by addition of 30 μl of a 1:1 suspension of protein G-Sepharose (Incospharm Corporation), after which the mixture was incubated for 2 h at 4 °C with gentle rotation. The beads were pelleted and washed three times with lysis buffer (20 mM HEPES-NaOH pH 7.5, 0.15 M NaCl, 2 mM CaCl_2_, 2 mM MgCl_2_, 1% Triton X-100, 0.2 mM PMSF, 1 μg/ml aprotinin, 1 μg/ml leupeptin, 1 μg/ml pepstatin, 1 mM Na_3_VO_4_). Immune complexes were then resolved by sodium dodecyl sulfate-polyacrylamide gel electrophoresis (SDS-PAGE) and immunoblotted with anti-PSD-95, anti-PSD-93, anti-SAP102, anti-S-SCAM, anti-CASK, anti-Shank3, and anti-Slitrk2 antibodies. Human embryonic kidney 293 T (HEK293T) cells were maintained in Dulbecco’s Modified Eagle’s medium (DMEM) containing 10% fetal bovine serum (FBS) and 100 U/ml of penicillin-streptomycin. HEK293T cells were then transfected with the indicated combination of plasmids. After 48 h, the transfected HEK293T cells were rinsed with ice-cold phosphate-buffered saline (PBS) and solubilized in lysis buffer (20 mM Tris pH 7.4, 1.0% Triton X-100, 0.1% SDS, 150 mM NaCl, 10% glycerol, 0.2 mM PMSF, 1 μg/ml aprotinin, 1 μg/ml leupeptin, 1 μg/ml pepstatin, 1 mM Na_3_VO_4_). After centrifugation at 20,000 × g, the supernatants were incubated with 1 μg of the appropriate antibody overnight at 4 °C. Thereafter, 30 μl of a 1:1 suspension of protein A-Sepharose (Incospharm Corporation) was added, and the mixture was incubated for 2 h at 4 °C with gentle rotation. Immune complexes were then resolved by SDS-PAGE and immunoblotted with the indicated antibodies. Coimmunoprecipitation experiments were repeated at least three times, and quantified results are expressed as the amount of protein co-precipitated relative to input amount. Representative immunoblot images are presented in the indicated figures.

### Co-clustering assay in COS-7 cells

Co-clustering assays were performed as previously described^17^. Briefly, COS-7 cells doubly transfected with HA-tagged Slitrk2 and GW1-PSD-95 or EGFP-Shank3a were fixed with 4% paraformaldehyde, permeabilized with 0.2% Triton X-100, and immunostained using anti-PSD-95, anti-EGFP (enhanced green fluorescent protein) and anti-HA (hemagglutinin) antibodies, followed by immunofluorescence staining with FITC- or Cy3-conjugated secondary antibodies (Jackson ImmunoResearch). Images were acquired by confocal microscopy (LSM800; Zeiss)

### Primary neuron culture, transfection, immunocytochemistry, and quantitative analyses

Cultured hippocampal neurons were prepared from embryonic day 18 (E18) rat brains, as previously described^18^. Briefly, hippocampal neurons were prepared from E18 rat brains and cultured on coverslips coated with poly-D-lysine in Neurobasal media supplemented with B-27 (Thermo-Fisher), 0.5% fetal bovine serum, 0.5 mM Glutamax (Thermo-Fisher), and sodium pyruvate (Thermo-Fisher). For immunocytochemistry, neurons were fixed with 4% paraformaldehyde/sucrose, permeabilized with 0.1% Triton X-100 in PBS, incubated with the indicated primary antibodies, and detected with Cy3- and FITC-conjugated secondary antibodies (Jackson ImmunoResearch). The indicated primary antibodies against the following proteins were used for immunocytochemical analyses of transfected neurons: EGFP (Rockland; 1:700), HA (Covance; 1:500), PSD-95 (NeuroMab; 1:100), VGLUT1 (Synaptic Systems; 1:700), GAD65 (JK158; 1:300), and Shank (1172; 1:200). For overexpression of Slitrk2, neurons at 10 days *in vitro* (DIV10) were transfected with pDisplay hSlitrk2 or pDisplay hSlitrk2 ΔPBM together with a L-315 vector using a CalPhos Kit (Clontech), and then immunostained at DIV14. For knockdown (KD) of Slitrk2, cultured neurons were transfected with L-315 alone (Control), L-315 sh-Slitrk2 (Slitrk2-KD), or cotransfected with L-315 sh-Slitrk2 and pDisplay-hSlitrk2 vector (WT Slitrk2 rescue) or pDisplay hSlitrk2 ΔPBM (Slitrk2 ΔPBM rescue) at DIV8 and immunostained at DIV14. Fluorescence images were acquired using a confocal laser-scanning microscope (LSM800, Carl Zeiss) with a 63x objective lens. Image-acquisition settings were kept constant during scanning. Z-stack images were converted to maximal projection and analyzed to obtain the size, intensity, and density of immunoreactive puncta corresponding to marker proteins. Two or three primary dendrites were selected and those within 100 μm of the first branched points were targeted for quantifications in a blinded manner using MetaMorph software (Molecular Devices). For purposes of dendritic spine quantification, a dendritic spine was defined as an EGFP-positive dendritic protrusion (0.4–3 μm in length).

### Production of recombinant adeno-associated virus (AAV)

HEK293T cells were co-transfected with the indicated AAV vectors and pHelper and AAV1.0 (serotype2/9) capsids vectors. Seventy-two hours later, transfected HEK293T cells were collected, lysed, and mixed with 40% polyethylene glycol and 2.5 M NaCl, and centrifuged at 2000 × g for 30 min. The cell pellets were resuspended in HEPES buffer (20 mM HEPES; 115 mM NaCl, 1.2 mM CaCl_2_, 1.2 mM MgCl_2_, 2.4 mM KH_2_PO_4_) and an equal volume of chloroform was added. The mixture was centrifuged at 400 × g for 5 min, and concentrated three times with a Centriprep centrifugal filter (Millipore) at 1,220 × g for 5 min each and with an Amicon Ultra centrifugal filter (Millipore) at 16,000 × g for 10 min. Before titering AAVs, contaminating plasmid DNA was eliminated by treating 1 μl of concentrated, sterile-filtered AAVs with 1 μl of DNase I (Sigma-Aldrich) for 30 min at 37 °C. After treatment with 1 μl of stop solution (50 mM ethylenediaminetetraacetic acid) for 10 min at 65 °C, 10 μg of protease K (Sigma-Aldrich) was added and AAVs were incubated for 1 h at 50 °C. Reactions were inactivated by incubating samples for 20 min at 95 °C. The final virus titer was measured by quantitative reverse transcription-PCR (qRT-PCR) detection of *EGFP* sequences and subsequent reference to a standard curve generated using the pAAV-U6-EGFP plasmid. All plasmids were purified using a Plasmid Maxi Kit (Qiagen GmbH).

### Stereotaxic injection of rAAVs into mice

Five-week-old C57BL/6 mice were anesthetized by intraperitoneal injection of Avertin (400 mg/kg body weight). Viral solutions (titers ≥ 1 × 10^11^ viral genomes/ml) were injected with a NanoFil syringe (World Precision Instruments) at a flow rate of 0.1 μl/min. The coordinates used for the CA1 region of the dorsal hippocampus were AP −2.5 mm, ML ± 1.5 mm, DV + 1.5 mm (from the dura). The site at DV + 1.5 mm received a 1-μl injection. Injected mice were allowed to recover for at least 14 d following surgery prior to use in experiments.

### Immunohistochemistry

Mice were transcardially perfused first with PBS and then with 4% paraformaldehyde. After post-fixation overnight, mouse brains were slowly sectioned at 40 μm using a vibratome (VT1200S; Leica) and washed with PBS. Brain sections were incubated in blocking solution containing 10% horse serum, 0.2% bovine serum albumin, and 2% Triton X-100 for 1 h at room temperature (RT), and then incubated overnight at 4 °C with primary antibodies against VGLUT1 (JK111; 1:200) or GAD65 (JK158; 1:200). After washing three times, sections were incubated with Cy3- or FITC-conjugated secondary antibodies (Jackson ImmunoResearch, West Grove, PA, USA) for 2 h at RT. Sections were then washed extensively and mounted on glass slides with Vectashield Mounting Medium (Vector Laboratories). Images were acquired using a confocal laser-scanning microscope (LSM700; Carl Zeiss).

### Electrophysiology

Whole-cell voltage-clamp recordings were performed in acute mouse brain slices, as previously described^15^. Mouse brain slices were transferred to a recording chamber and perfused with a bath solution of aerated (O_2_ 95%/CO_2_ 5% mixed gas) artificial cerebrospinal fluid (aCSF) consisting of 124 mM NaCl, 3.3 mM KCl, 1.3 mM NaH_2_PO_4_, 26 mM NaHCO_3_, 3.125 mM CaCl_2_, 2.25 mM MgCl_2,_ and 11 mM D-glucose at 25.4–25.7 °C. Glass pipettes (2–5MΩ) were filled with an intracellular solution containing the following: 145 mM CsCl, 5 mM NaCl, 10 mM HEPES, 10 mM EGTA, 4 mM Mg-ATP, and 0.3 mM Na-GTP. The osmolality of intracellular solution was 290–300 mOsm and the pH was 7.3 (adjusted with CsOH). For mEPSC recordings, 1 μM TTX (Tocris) and 50 μM picrotoxin (Tocris) added to bath solution to block Na^+^ currents and GABA_A_ receptors. For mIPSC recordings, 1 μM TTX, 10 μM CNQX (Sigma Aldrich) and 50 μM D-AP-5 (Tocris) added to block Na^+^ currents, AMPAR and NMDAR.

### Statistical analysis

All data are presented as means ± SEM. All experiments were repeated using at least three independent cultures, and data were statistically evaluated using a Mann-Whitney U test or Kruskal-Wallis test followed by Dunn’s pairwise *post hoc* test, as appropriate. Prism8.0 (GraphPad Software) was used for analysis of data and preparation of bar graphs. P-values < 0.05 were considered statistically significant (individual *p*-values are presented in respective figure legends).

## Results

### The C-terminal PDZ domain-binding sequence in Slitrk2 mediates interactions with PSD-95 and Shank3

**Figure 1:**
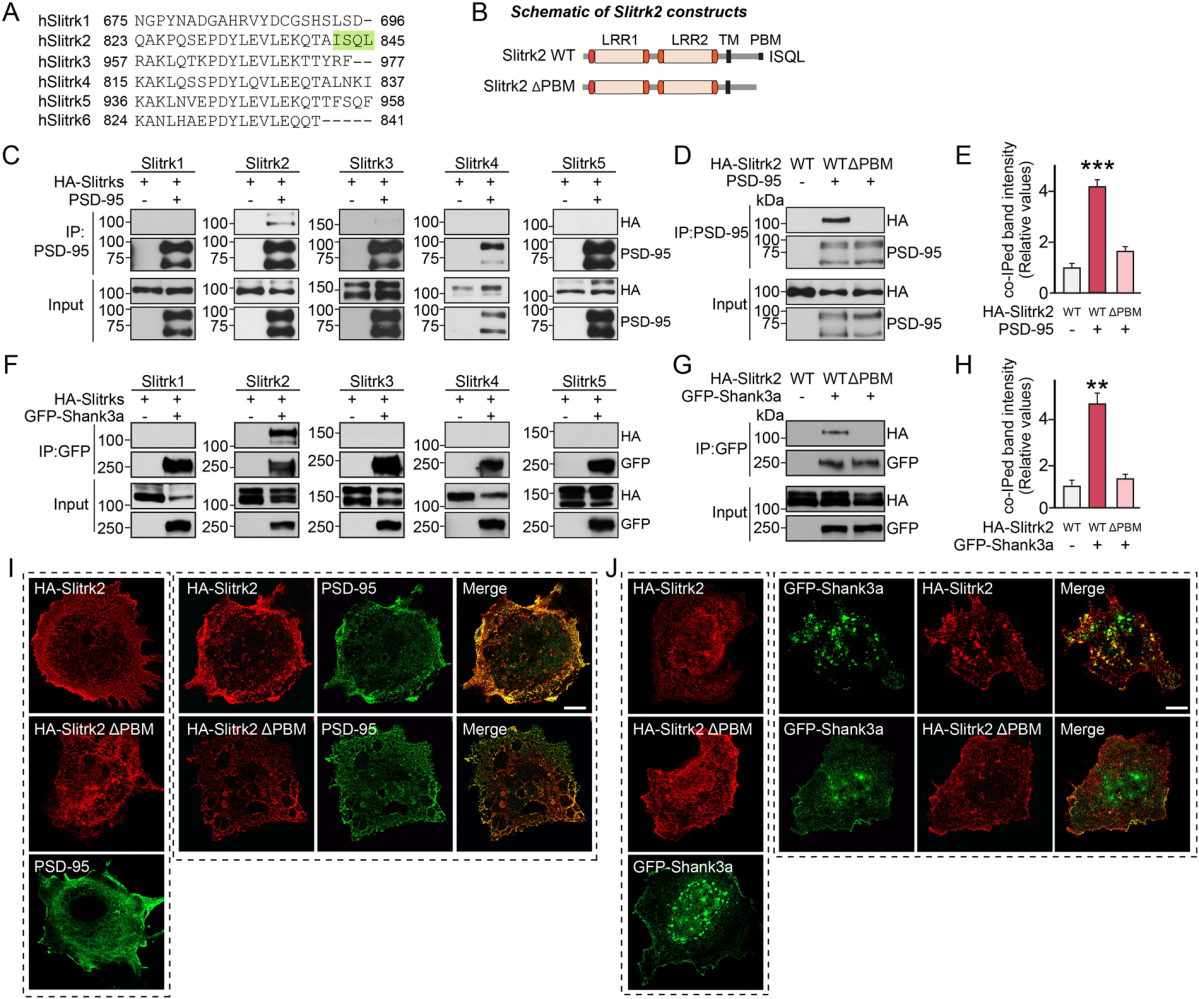
Slitrk2, but not other Slitrks, interacts with PSD-95 and Shank3 via its C-terminal PDZ binding motif. (**A**) C-terminal amino acid sequences of human Slitrks. The green box indicates the PDZ-binding motif found only in Slitrk2. (**B**) Schematic diagrams of Slitrk2 WT and a Slitrk2 mutant lacking a C-terminal PDZ-binding motif (Slitrk2 ΔPBM). Abbreviation: PBM, PDZ-binding motif. (**C**) Coimmunoprecipitation experiment demonstrating that Slitrk2, but not other Slitrks, interacts with PSD-95. HEK293T cells were transfected with HA-tagged Slitrk1, Slitrk2, Slitrk3, Slitrk4 or Slitrk5, alone or together with PSD-95, after which coimmunoprecipitation of Slitrks with PSD-95 was assayed. Input, 5%. (**D**) Coimmunoprecipitation experiment showing that the C-terminal PDZ-binding motif of Slitrk2 is necessary for interaction with PSD-95. HEK293T cells were transfected with HA-tagged Slitrk2 or Slitrk2 ΔPBM together with PSD-95, after which coimmunoprecipitation of Slitrk2 with PSD-95 was assayed. Input, 5%. (**E**) Quantification of coimmunoprecipitated Slitrk2 in (**D**), normalized to control. Data are means ± SEM from three independent experiments. (^***^p < 0.001; non-parametric Kruskal-Wallis test with Dunn’s *post-hoc* test). (**F**) Coimmunoprecipitation experiment demonstrating that Slitrk2, but not other Slitrks, interacts with Shank3a. HEK293T cells were transfected with HA-tagged Slitrk1, Slitrk2, Slitrk3, Slitrk4 or Slitrk5, alone or together with EGFP-tagged Shank3a, after which coimmunoprecipitation of Slitrks with Shank3a was assayed. Input, 5%. (**G**) Coimmunoprecipitation experiment showing that the C-terminal PDZ-binding motif of Slitrk2 is necessary for interaction with Shank3a. HEK293T cells were transfected with HA-tagged Slitrk2 or Slitrk2 ΔPBM together with Shank3a, after which coimmunoprecipitation of Slitrk2 with Shank3a was assayed. Input, 5%. (**H**) Quantification of coimmunoprecipitated Slitrk2 levels in (**G**), normalized to control. Data are means ± SEM from three independent experiments. (^**^p < 0.01; non-parametric Kruskal-Wallis test with Dunn’s *post hoc* test). (**I** and **J**) Representative images of COS-7 cells transfected with HA-Slitrk2, HA-Slitrk2ΔPBM, PSD-95 (**I**) or EGFP-Shank3a (**J**) alone, or HA-Slitrk2 cotransfected with PSD-95 (**I**), HA-Slitrk2 ΔPBM cotransfected with PSD-95 (**I**), HA-Slitrk2 cotransfected with EGFP-Shank3a (**J**), or HA-Slitrk2 ΔPBM cotransfected with EGFP-Shank3a (**J**). Scale bar, 10 µm (applies to all images).

Among the six Slitrks, only Slitrk2 contains a canonical type-I PDZ domain-binding motif at its C-terminus that recognizes the PDZ domain^19^ (Fig. 1A), raising the possibility that Slitrk2 interacts with intracellular PDZ domain-containing scaffolds. To test this possibility, we performed co-immunoprecipitation analyses in HEK293T (human embryonic kidney 293T) cells expressing the indicated Slitrk isoforms (Slitrk1–5) alone or coexpressing Slitrks and PSD-95 or Shank3. We found that Slitrk2 robustly and specifically interacted with PSD-95 and Shank3 (Figs. 1C,F, S1A,C). These interactions were completely disrupted by deleting the last four amino acids residues of Slitrk2 (Ile-Ser-Glu-Leu; ISQL) (Figs. 1B,D,E,G,H, S1B,D), suggesting a canonical PDZ domain-mediated interaction between Slitrk2 and the tested PDZ domain-containing proteins.

**Figure 2:**
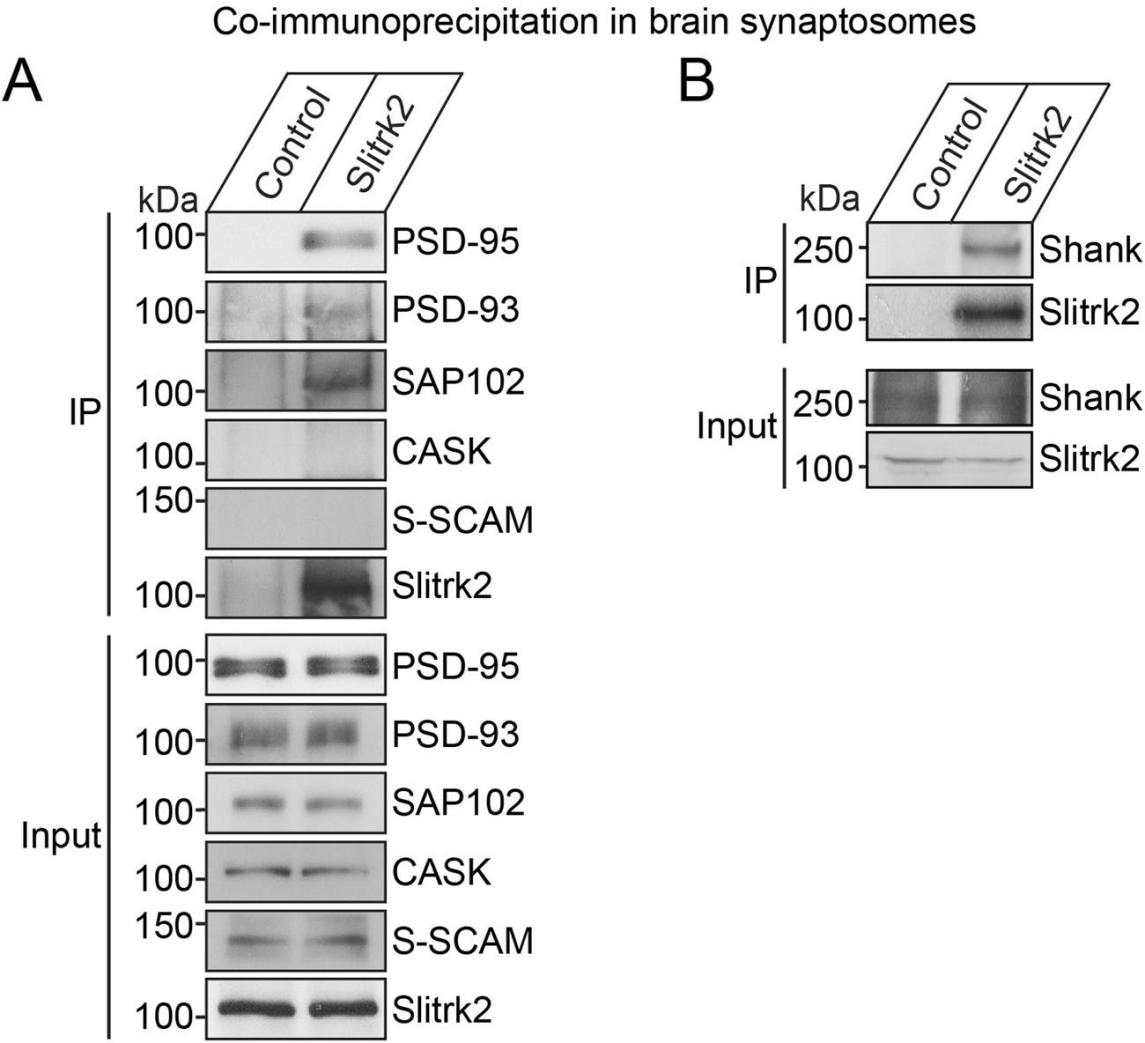
Slitrk2 forms complexes with PSD-95 and Shank *in vivo*. (**A**) Coimmunoprecipitation experiment in mouse brain lysates demonstrating that Slitrk2 forms complexes with PSD-95, SAP102 or PSD-93, but not with CASK or S-SCAM. Synaptosomal fractions of adult mouse brains were immunoprecipitated with anti-Slitrk2 antibodies and immunoblotted with the indicated antibodies. An equal amount of rabbit IgG (Control) was used as a negative control. Input, 5%. (**B**) Synaptosomal fractions of adult mouse brains were immunoprecipitated with anti-Slitrk2 antibodies and immunoblotted with anti-pan-Shank antibodies. An equal amount of rabbit IgG (Control) was used as a negative control. Input, 5%.

To corroborate these findings, we performed co-clustering analyses using non-neuronal COS-7 cells. When singly expressed, Slitrk2 and PSD-95 were evenly distributed in the cytoplasm of transfected COS-7 cells (Fig. 1I), whereas Shank3 uniquely exhibited a nuclear localization^20^ (Fig. 1J). However, coexpression of Slitrk2 and PSD-95 or Shank3 in COS-7 cells led to the formation of colocalized clusters in the cytoplasm, consistent with the direct interaction of Slitrk2 with PSD-95 or Shank3 (Fig. 1I,J). Lastly, we found that Slitrk2, immunoprecipitated from crude synaptosome lysates of adult rat brains with Slitrk2 antibodies, coimmunoprecipitated PSD-95, PSD-93, SAP102, Shank, but not S-SCAM and CASK (Figs. 2A,B, S2A,B). Taken together, these results indicate that Slitrk2 uniquely interacts with PDZ domain-containing scaffolds *in vitro* and *in vivo*.

### PDZ1 + 2 domains of PSD-95 and a single PDZ domain of Shank3 mediate binding to Slitrk2

**Figure 3:**
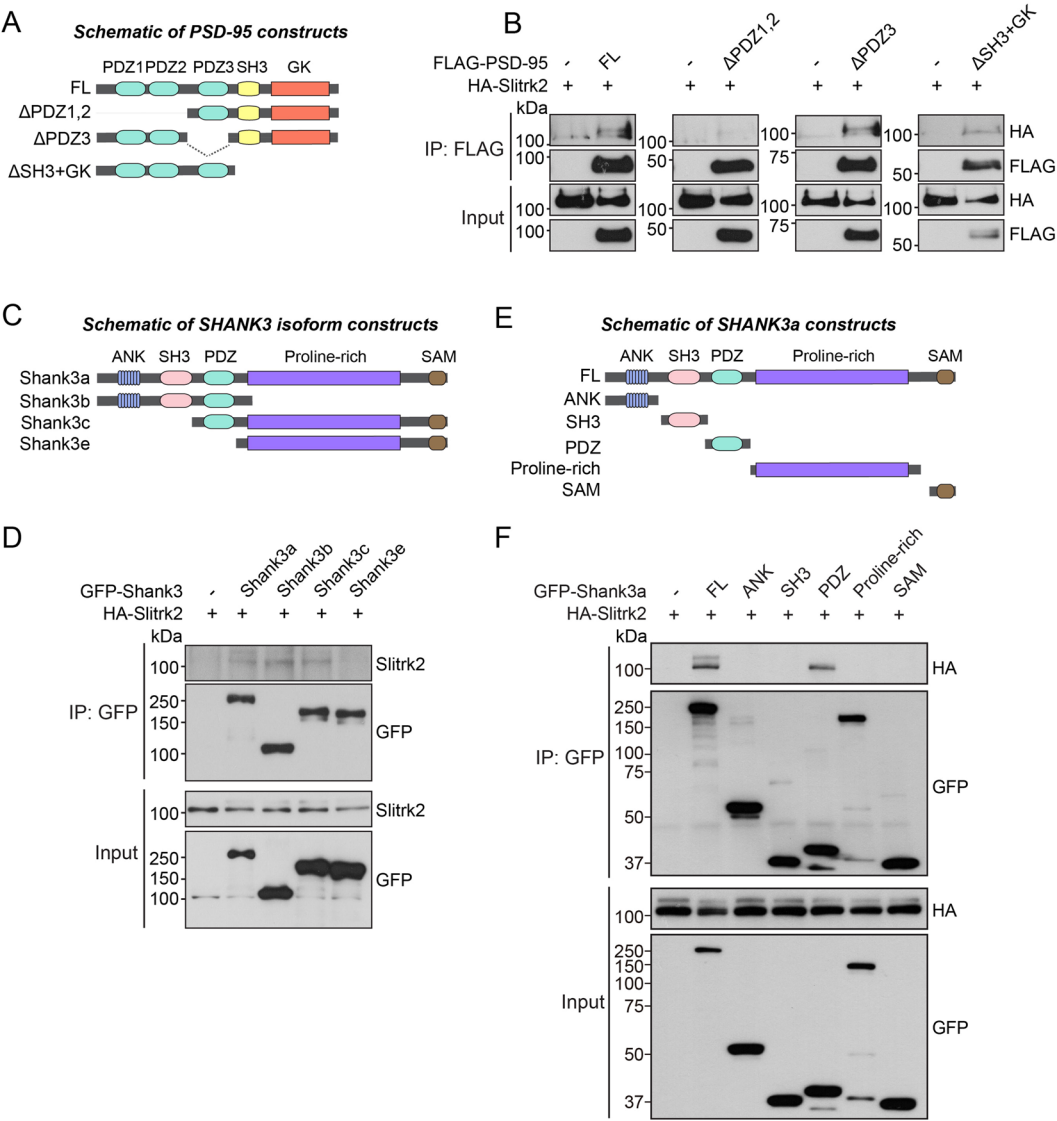
The C-terminal PDZ-binding motif of Slitrk2 is required for Slitrk2 binding to PDZ domains of PSD-95 or the PDZ domain of Shank3a. (**A**) Schematic diagrams of a series of PSD-95 deletion constructs. Abbreviation: PDZ, PSD-95/Dlg-A/ZO-1; SH3, src homology 3; and GK, guanylate kinase. (**B**) Coimmunoprecipitation experiment demonstrating that PDZ1 + 2 domains of PSD-95 are required for interaction with Slitrk2. HEK293T cells were transfected with HA-tagged Slitrk2 alone or together with the indicated PSD-95 deletion constructs, after which coimmunoprecipitation of Slitrk2 with PSD-95 was assayed. Input, 5%. (**C**) Schematic diagrams of Shank3a, Shank3b, Shank3c, and Shank3e expression vectors. Abbreviations: ANK, ankyrin repeat; PDZ, PSD-95/Dlg-A/ZO-1; SH3, Src homology 3; and SAM, sterile alpha motif. (**D**) HEK293T cells were transfected with HA-tagged Slitrk2 alone or together with Shank3 variants (**C**), after which coimmunoprecipitation of Slitrk2 with Shank3 was assayed. Input, 5%. (**E**) Schematic diagrams of a series of Shank3a deletion constructs. Abbreviations: ANK, ankyrin repeat; PDZ, PSD-95/Dlg-A/ZO-1; SH3, Src homology 3; and SAM, sterile alpha motif. (**F**) Coimmunoprecipitation experiment demonstrating that the PDZ domain of Shank3a is sufficient for interaction with Slitrk2. HEK293T cells were transfected with HA-tagged Slitrk2 alone or together with Shank3a deletion constructs, after which coimmunoprecipitation of Slitrk2 with Shank3a was assayed. Input, 5%.

To determine the regions in PSD-95 and Shank3 responsible for interacting with Slitrk2, we generated a series of PSD-95 and Shank3 expression constructs and performed coimmunoprecipitation experiments in HEK293T cells. Three deletion variants of PSD-95, denoted “Full, ΔPDZ1 + 2, ΔPDZ3, and ΔGK-SH3”, five deletion variants of Shank3a, denoted “Full, ANK, SH3, PDZ, Proline-rich, and SAM”, and four different splice variants of Shank3, denoted “Shank3a–e”^13^, were tested (Fig. 3A,C,E). As expected, we found that Slitrk2 interacted with PDZ1 + 2 domain-containing fragments of PSD-95, PDZ domain-containing Shank3 isoforms (Shank3a, Shank3b, and Shank3c) and PDZ domain-containing fragments of Shank3a, but not with Shank3e, which lacks the PDZ domain (Figs. 3B,D,F, S3).

### The C-terminal PDZ domain-binding sequence of Slitrk2 is required for Slitrk2 promotion of excitatory synapse development in cultured hippocampal neurons

**Figure 4:**
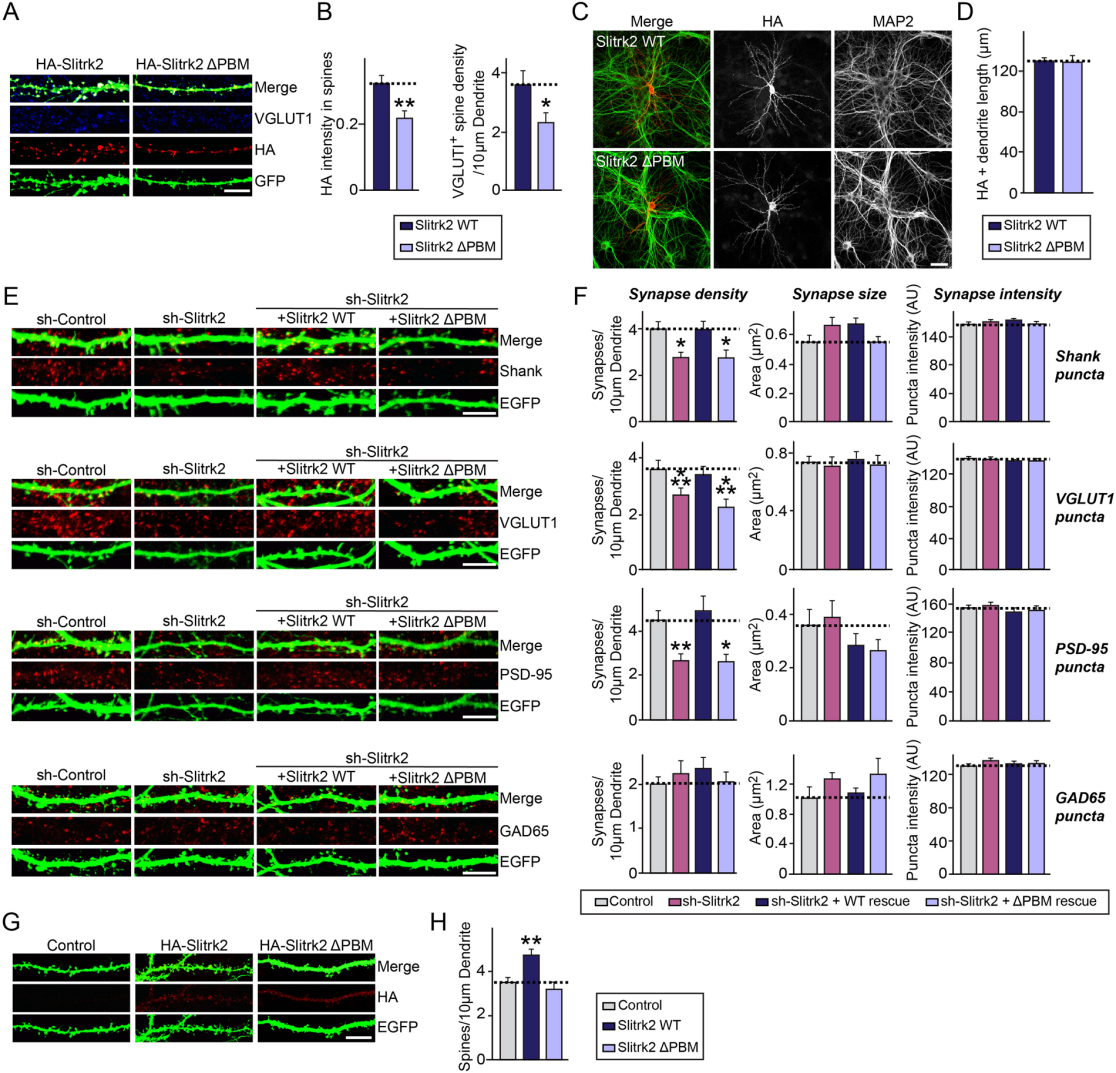
Slitrk2 promotes excitatory synapse development through its C-terminal PDZ interactions in cultured hippocampal neurons. (**A**) Representative images of cultured hippocampal neurons transfected at DIV10 with Slitrk2 constructs (Slitrk2 WT or Slitrk2 ΔPBM). Neurons were analyzed by triple-immunofluorescence labeling for VGLUT1 (blue), HA (red), and MAP2 (green) at DIV14. Scale bar, 10 μm (applies to all images). (**B**) Summary bar graphs showing the intensity of HA immunofluorescence-positive spines (left) and the number of VGLUT1 immunofluorescence-positive spines (right). Data are presented as means ± SEMs from three independent experiments (n = 22–30 neurons; *p < 0.05, **p < 0.01 vs. control; non-parametric Mann-Whitney U test). (**C**) Representative images of cultured hippocampal neurons transfected at DIV10 with Slitrk2 constructs (Slitrk2 WT or Slitrk2 ΔPBM). Neurons were analyzed by double-immunofluorescence labeling for HA (red) and MAP2 (green) at DIV14. Scale bar, 20 μm (applies to all images). (**D**) Summary data for lengths of HA immunofluorescence-positive dendrites. Data are presented as means ± SEMs from three independent experiments (n = 22–30 neurons; non-parametric Mann-Whitney U test). (**E**) Cultured hippocampal neurons were transfected with a lentiviral vector expressing sh-Control, sh-Slitrk2, or coexpressing sh-Slitrk2 and shRNA-resistant Slitrk2 expression vectors (Slitrk2 WT or Slitrk2 ΔPBM) at DIV8 and analyzed at DIV14 by double-immunofluorescence staining for EGFP (green) and the indicated synaptic markers (Shank, VGLUT1, PSD-95 or GAD65; red). Scale bar, 10 μm (applies to all images). (**F**) Summary data showing the effects of Slitrk2 KD on synaptic puncta density (**left**) and synaptic puncta size (**right**), measured using Shank, VGLUT1, PSD-95, and GAD65 as synaptic markers. More than three dendrites per transfected neuron were analyzed and group-averaged. Data are presented as means ± SEMs from three independent experiments (n = 22–30 neurons; *p < 0.05, **p < 0.01, ***p < 0.001 vs. control; non-parametric ANOVA with Kruskal-Wallis test followed by *post hoc* Dunn’s multiple comparison test). (**G**) Representative images of cultured hippocampal neurons transfected at DIV10 with EGFP alone (Control) or together with Slitrk2 constructs (Slitrk2 WT or Slitrk2 ΔPBM). Neurons were analyzed by double-immunofluorescence labeling for HA (red) and EGFP (green) at DIV14. Scale bar, 10 μm (applies to all images). (**H**) Summary data showing the effects of Slitrk2 overexpression in neurons on dendritic spine density. Data are presented as means ± SEMs from three independent experiments (n = 22–30 neurons; **p < 0.01 vs. control; non-parametric ANOVA with Kruskal-Wallis test followed by *post hoc* Dunn’s multiple comparison test).

To investigate the functional significance of PDZ domain-mediated interactions for Slitrk2 targeting to excitatory synapses, we first transfected cultured hippocampal neurons at DIV10 with expression vectors encoding HA-tagged Slitrk2 wild-type (WT) and a Slitrk2 variant lacking the C-terminal four amino acids (ΔPBM), and immunostained the transfected neurons for the excitatory presynaptic marker VGLUT1 (vesicular glutamate transporter 1) at DIV14. Investigation of the subcellular localization of recombinant Slitrk2 variants, visualized by monitoring expression of HA immunofluorescence, revealed that Slitrk2 WT was mainly distributed to dendritic spines, whereas recombinant Slitrk2 ΔPBM showed less distribution to dendritic spines compared with Slitrk2 WT (Fig. 4A,B). In addition, the number of VGLUT1-positive dendritic spines was reduced following overexpression of recombinant Slitrk2 ΔPBM, although both Slitrk2 WT and ΔPBM exhibited comparable levels of dendritic targeting when expressed in mature cultured hippocampal neurons (Fig. 4A–D).

Next, to determine whether Slitrk2 ΔPBM also compromises the ability of Slitrk2 WT to promote excitatory synapse development in cultured hippocampal neurons, we introduced Slitrk2-specific knockdown (KD) vectors into cultured neurons at DIV8, and immunostained the transfected neurons for EGFP and various excitatory synaptic markers (VGLUT1, PSD-95, and pan-Shank) at DIV14 (Fig. 4E). As previously reported^10,12^, Slitrk2 KD significantly decreased the linear density of excitatory synaptic clusters (Fig. 4E,F). Coexpression of short hairpin RNA (shRNA)-resistant forms of Slitrk2 WT completely rescued these deficits in the numbers of excitatory synaptic clusters (Fig. 4E,F). However, coexpression of shRNA-resistant Slitrk2 ΔPBM failed to reverse the reduction in the density of excitatory synaptic clusters induced by Slitrk2 KD (Fig. 4E,F). Moreover, and in keeping with our previous observations^10,12^, Slitrk2 KD significantly decreased the number of dendritic spines, which was completely rescued by coexpression of Slitrk2 WT, but not by coexpression of Slitrk2 ΔPBM (Fig. S4). In addition, overexpression of Slitrk2 WT led to a significant increase in the number of dendritic spines in transfected neurons (Fig. 4G,H), whereas overexpression of Slitrk2 ΔPBM did not (Fig. 4G,H). These data suggest that Slitrk2 promotes the development of excitatory synapses in cultured hippocampal neurons via PDZ domain-mediated interactions.

### Slitrk2 promotes excitatory synapse structure and transmission through its C-terminal PDZ-mediated interactions in CA1 hippocampal pyramidal neurons *in vivo*

**Figure 5:**
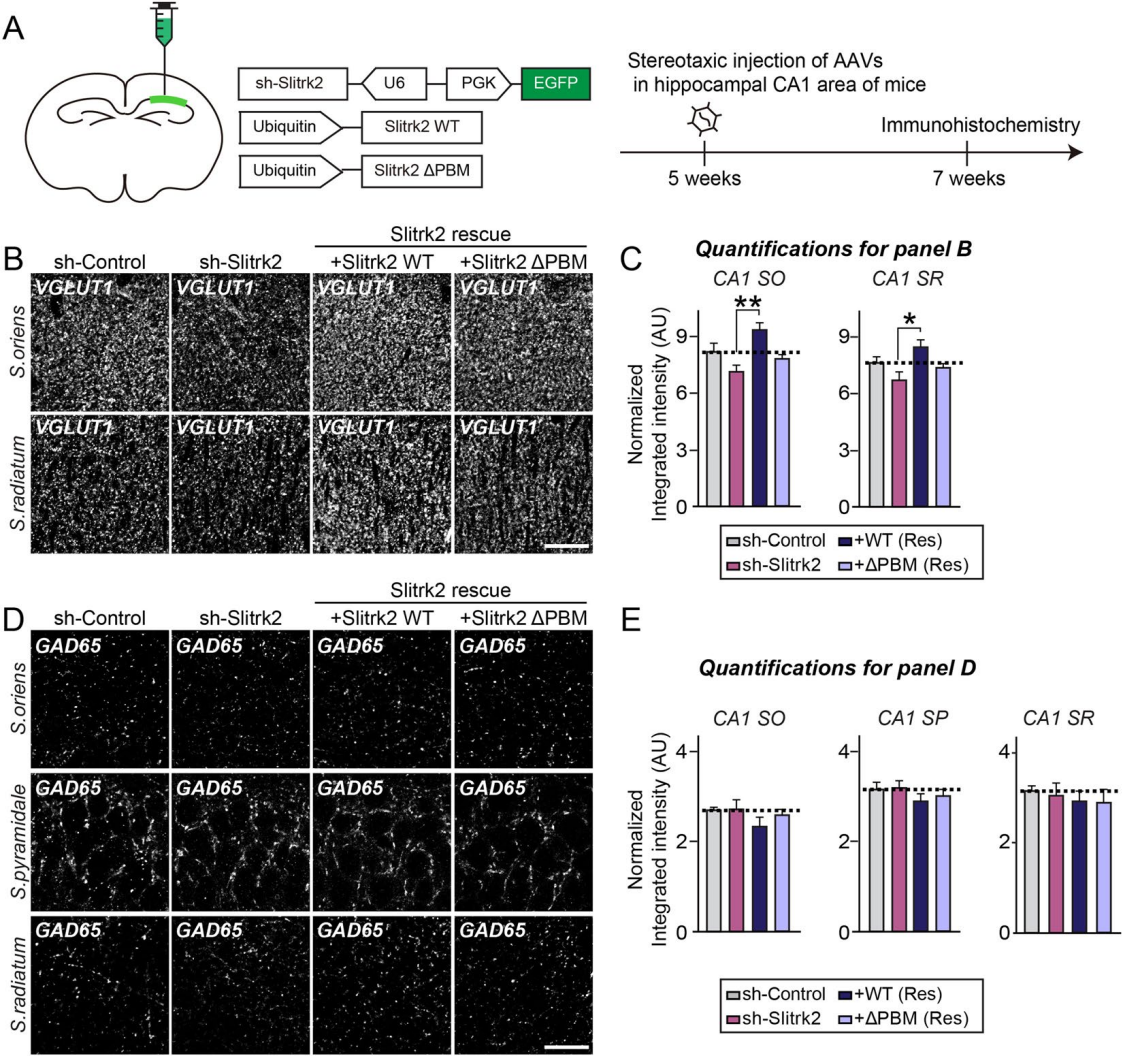
Slitrk2 acts through its C-terminal PDZ-mediated interactions to promote excitatory synapse development in CA1 hippocampal pyramidal neurons *in vivo*. (**A**) Experimental design for immunohistochemical analyses. The CA1 region of the hippocampus of ~5-wk-old WT mice was bilaterally injected with the indicated AAVs. AAV-injected mice were subjected to immunohistochemical analyses 2 wk after injections. (**B**) Representative images of hippocampal CA1 SO and SR regions 2 wk after stereotactic injection of the indicated AAVs into WT mice, followed by immunostaining for the excitatory synapse marker VGLUT1. Scale bar: 20 μm (applies to all images). (**C**) Quantification of the integrated intensity of VGLUT1-positive synaptic puncta. Data are means ± SEM (*p < 0.05; non-parametric Kruskal-Wallis test with Dunn’s *post hoc* test; n = 4–5 mice each after averaging data from 3 sections/mouse). (**D**) Representative images of hippocampal CA1 SO, SP, and SR regions 2 wk after stereotactic injections of the indicated AAVs into WT mice, followed by immunostaining for the inhibitory synapse marker GAD65. Scale bar: 20 μm (applies to all images). (**E**) Quantification of the integrated intensity of GAD65-positive synaptic puncta. Data are means ± SEMs (non-parametric Kruskal-Wallis test with Dunn’s *post hoc* test; n = 4–5 mice each after averaging data from 3 sections/mouse).

To test the physiological significance of Slitrk2 PDZ domain-mediated interactions *in vivo*, we performed immunohistochemistry using mice stereotactically injected with adeno-associated viruses (AAVs) expressing Slitrk2-targeting shRNA (sh-Slitrk2) or non-targeting control shRNA (sh-Control) into the hippocampal CA1 areas (Figs. 5A, S5A). Slitrk2 KD *in vivo* was validated by immunohistochemistry and immunoblotting analyses using the indicated AAV-injected mouse brain tissues (Fig. S5B,C). Quantitative immunofluorescence analyses using the excitatory synaptic marker VGLUT1 revealed a slight (but statistically non-significant) reduction in the intensity of VGLUT1 puncta in both stratum oriens (SO) and stratum radiatum (SR) layers of the hippocampal CA1 (Fig. 5B,C). In contrast, the intensity of GABAergic synaptic marker GAD65 (glutamic acid decarboxylase 65) puncta was not altered in Slitrk2-deficient neurons (Fig. 5D,E). Intriguingly, the VGLUT1 puncta intensity in Slitrk2-KD mice was increased by coexpression of shRNA-resistant Slitrk2 WT, but not by coexpression of shRNA-resistant Slitrk2 ΔPBM, despite the fact that both Slitrk2 WT and Slitrk2 ΔPBM exhibited comparable expression levels (Figs. 5B,C, S5B,C).

**Figure 6:**
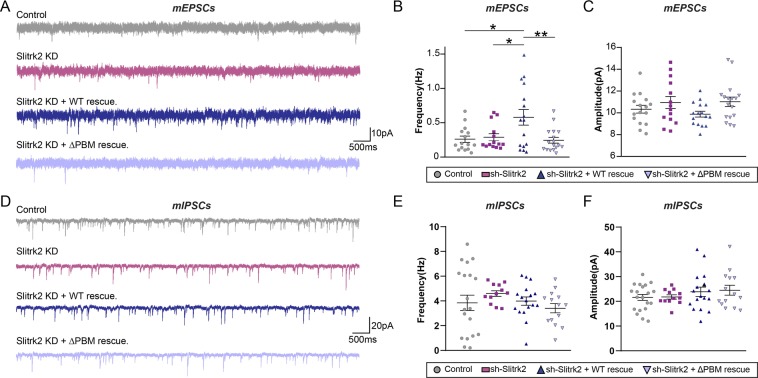
Slitrk2 acts through its C-terminal PDZ-mediated interactions to promote excitatory synaptic transmission in CA1 hippocampal pyramidal neurons *in vivo*. (**A–C**) Representative traces (**A**) and summary graphs showing the frequency (**B**) and amplitude (**C**) of mEPSCs recorded from hippocampal CA1 pyramidal neurons infected with the indicated AAVs. Bar graphs show means ± SEMs (*p < 0.05, **p < 0.01; non-parametric Kruskal-Wallis test with Dunn’s *post hoc* test; ‘n’ denotes the total number of neurons analyzed as follows: Ctrl, n = 15 cells from 8 mice; Slitrk2 KD, n = 14 cells from 7 mice; Slitrk2 KD + WT rescue, n = 16 cells from 6 mice; Slitrk2 KD + ΔPBM rescue, n = 17 cells from 7 mice). (**D–F**) Representative traces (**D**) and summary graphs showing the frequency (**E**) and amplitude (**F**) of mIPSCs recorded from hippocampal CA1 pyramidal neurons infected with the indicated AAVs. Graphs show means ± SEMs (non-parametric Kruskal-Wallis test with Dunn’s *post hoc* test; ‘n’ denotes the total number of neurons analyzed as follows: Ctrl, n = 20 cells from 5 mice; Slitrk2 KD, n = 12 cells from 5 mice; Slitrk2 KD + WT rescue, n = 17 cells from 5 mice; Slitrk2 KD + ΔPBM rescue, n = 14 cells from 4 mice).

To corroborate these anatomical findings, we performed whole-cell electrophysiological recordings of miniature excitatory and inhibitory postsynaptic currents (mEPSCs and mIPSCs, respectively) in acutely prepared mouse brain slices infected with adeno-associated viruses (AAVs) expressing either sh-Slitrk2 or sh-Control. Consistently, we did not detect any noticeable difference in the amplitude or frequency of mEPSCs or mIPSCs in Slitrk2-deficient neurons (Fig. 6A–F)^10^. Again, coexpression of Slitrk2 WT in Slitrk2-deficient neurons led to a significant increase in the frequency (but not amplitude) of mEPSCs, whereas coexpression of Slitrk2 ΔPBM did not (Fig. 6A–C). Interestingly, the frequency of mEPSCs under these conditions exceeded that in controls, a result that may be an artifact of Slitrk2 overexpression reflecting the high level expression of Slitrk2 WT AAV constructs. Taken together, our data suggest that Slitrk2 is not required for basal synaptic transmission at either synapse type *in vivo*, and that Slitrk2 specifically regulates excitatory synapse organization in hippocampal CA1 neurons through interactions with PDZ domain-containing proteins.

## Discussion

Recent efforts to identify a number of *trans*-synaptic adhesion molecules and investigate their synaptic roles has significantly contributed to our current understanding of how synapses are formed, refined, and eliminated^2^. The initial conceptualization of these molecules was as building blocks for structural organization, and some candidates in invertebrate synapses have been proposed in this context. However, in vertebrate synapses, this perspective has broadened to encompass all biological processes, including synaptic initiation, assembly, and organization of canonical signaling ensembles^2^. Strikingly, the roles of most trans-synaptic adhesion molecules expressed in mammalian synapses as signaling entities have remained largely unknown.

PSD-95, by extension, the PSD-95 family of membrane-associated guanylate kinases (MAGUKs), together with gephyrin are arguably the most extensively studied synaptic proteins that exclusively expressed in excitatory and inhibitory synaptic sites^21–25^. These proteins are structurally modular and form multimeric complexes with neurotransmitter receptors, synaptic adhesion molecules and downstream signaling effectors, serving as scaffolding cornerstones that mediate diverse structural and functional organization. Notably, PSD-95 and gephyrin contribute to the stabilization of interacting *trans*-membrane proteins (NMDA- and AMPA-type glutamate receptors, and various synaptic adhesion molecules) by suppressing their surface mobility or internalization^21^. Among the domain architectural features of MAGUKs, three PDZ domains are involved in recognizing specific C-terminal motifs present in a variety of *trans*-membrane proteins^26^. However, to date, only neuroligin-2 (expressed exclusively at GABAergic synaptic sites) has been reported to bind gephyrin, a uniqueness that may largely reflect the lack of an identifiable gephyrin-binding consensus sequence^23^. Thus, efforts to test the putative interactions of MAGUK PDZ domains with various *trans*-membrane proteins have proved fruitful for describing the molecular organization of excitatory synapses^25^. A number of putative *trans*-synaptic adhesion molecules have been subsequently shown to interact with MAGUK family proteins, including neuroligins (NLs)^27^, SALMs^28,29^, Netrin-G ligands (NGLs)^30^, ADAM22^31^, LRRTMs^15,32^, Kirrels/Nephs^33^, and IgSF11^34^. In most cases, however, the precise physiological roles of these PDZ domain-mediated interactions are still undefined. NLs are targeted to synaptic sites, independent of PDZ domain-mediated interactions^35^; for these proteins, targeting instead requires non-PDZ domain-mediated intracellular mechanisms (e.g., activity-dependent posttranslational modifications)^36^. In contrast, NGL-2 and IgSF11 require PSD-95 interactions for excitatory synapse localization, and mediate the physical and functional coupling of PSD-95 with other key components expressed at excitatory synaptic sites, such as AMPA-type glutamate receptors^30,34^. Although these observations suggest the compelling concept that PSD-95 matches synaptic adhesion molecules with various forms of intracellular signaling cascades to regulate excitatory synapse development in postsynaptic neurons, this hypothesis has not been systematically addressed.

Our current study sought to identify intracellular mechanisms governing the actions of Slitrks, an emerging class of postsynaptic adhesion molecules^4,7^. Taking note of the fact that Slitrk2 (among six Slitrk members) possesses a typical type I PDZ domain-binding motif at its C-terminus, we here showed that Slitrk2 directly interacts with two key PDZ domain-containing synapse organizers, PSD-95 and Shank3 (Figs. 1 and 2). We also demonstrated that Slitrk2 promotes excitatory synapse structure and transmission through these PDZ domain-mediated interactions *in vitro* and *in vivo* (Figs. 4–6). These observations suggest a tantalizing model in which interactions of Slitrk2 with PTPσ (a member of the LAR-RPTP family with specific functions at excitatory synapses)^5^ transduce/propagate extracellular information into specific intracellular complexes involving PSD-95, MAGUKs, and/or Shank3. However, our observation that other Slitrks do not interact with PSD-95 suggests that the molecular diversity of Slitrk-mediated synapse development depends on specific intracellular protein complexes (Fig. 1). Intriguingly, Slitrk2, -3 and -5 are similar to Trk receptors in that they contain a signature sequence motif (NpXY) at their juxtamembranous regions that usually serves as a binding site for adaptor proteins, such as Shc, following binding to specific neurotrophins and subsequent phosphorylation at a tyrosine residue^37^. Moreover, Slitrks contain various tyrosine residues that are conserved across all six members, hinting at the possibility that tyrosine phosphorylation could be an important regulatory mechanism for Slitrk-mediated synapse development. Prior studies have also demonstrated the significance of tyrosine phosphorylation of NL1 (Y782) in promoting recruitment of functional AMPA-type glutamate receptors and long-term potentiation, and excluding gephyrin binding^38,39^. Thus, tyrosine phosphorylation of synaptic adhesion molecules could be a universally important mechanism for establishing the balance between excitatory and inhibitory synapses during synapse formation, assembly, and stabilization.

Slitrk2 KD in cultured hippocampal neurons clearly reduced the number of excitatory synapses (Fig. 4^10,19^;), whereas Slitrk2 KD in mouse hippocampal CA1 regions did not significantly affect the density of excitatory synapses or excitatory synaptic transmission (Figs. 5 and 6). This apparent phenotypic difference between cultured neurons and *in vivo* neurons caused by the loss of Slitrk2 has been similarly documented in loss-of-function analyses of other synaptic proteins (e.g., neuroligins)^17,18,40^. Although the present study pinpointed a crucial contribution of the C-terminal PDZ-binding motif, which is unique to Slitrk2, in promoting excitatory synapse development, extracellular mechanisms shared by other excitatory synaptic Slitrks (i.e., Slitrk1, Slitrk4 and Slitrk5) may differentially compensate for the effect of Slitrk2 loss. Moreover, loss-of-function analyses of Slitrk2 were performed on sparsely transfected neurons, whereas AAV-mediated shRNA delivery downregulated Slitrk2 levels in most hippocampal CA1 neurons, suggesting the possibility that intercellular differences in Slitrk2 level are critical for excitatory synapse development, as previously reported for neuroligin-1, ephrin-B3 and TrkC^17,21,41^. These possibilities should be tested using conditional Slitrk2 mice to avoid possible developmental compensation.

Our demonstration that Slitrk2 interacts with Shank3 was unexpected, given the differential laminar organization of PSD scaffolds^42^. PSD-95 is spatially localized beneath PSD membranes, whereas Shank3 lies on the deeper cytoplasmic side of the PSD^42,43^. Because Slitrk2 has a relatively short cytoplasmic region (~203 residues), the direct interaction of Slitrk2 with Shank3 *in vivo* could be spatially constrained, despite our analyses showing robust interactions of Slitrk2 with Shank3 (Figs. 1 and 2). Future follow-up studies should address the differential roles of Slitrk2/PSD-95 and Slitrk2/Shank3 complexes in regulating excitatory synapse development *in vivo*.

## Supplementary information

Supplementary information

## Data Availability

The datasets generated and/or analyzed during the current study are available from the corresponding author upon request.
