# Supplementary material for: Slitrk2 controls excitatory synapse development via PDZ-mediated protein interactions
Source: Sci Rep. 2019 Nov 19;9:17094. doi: 10.1038/s41598-019-53519-1 (PMC6863843; doi:10.1038/s41598-019-53519-1)
Supplement: Supplementary file 1 — Supplementary information [file 41598_2019_53519_MOESM1_ESM.docx]

**Supplementary Information**

**Slitrk2 controls excitatory synapse development via PDZ-mediated protein interactions**

Kyung Ah Han^1^, Jinhu Kim^1^, Hyeonho Kim^1^, Dongwook Kim, Dongseok Lim, Jaewon Ko & Ji Won Um^#^

Department of Brain and Cognitive Sciences, Daegu Gyeongbuk Institute of Science and Technology (DGIST), 333 Techno Jungangdae-Ro, Hyeonpoong-eup, Dalseong-gun, Daegu 42988, Korea

^1^These authors contributed equally to this study

^#^Correspondence and requests for materials should be addressed to J.W.U. (email: [jiwonum@dgist.ac.kr](mailto:jiwonum@dgist.ac.kr))

**Supplementary Figures**


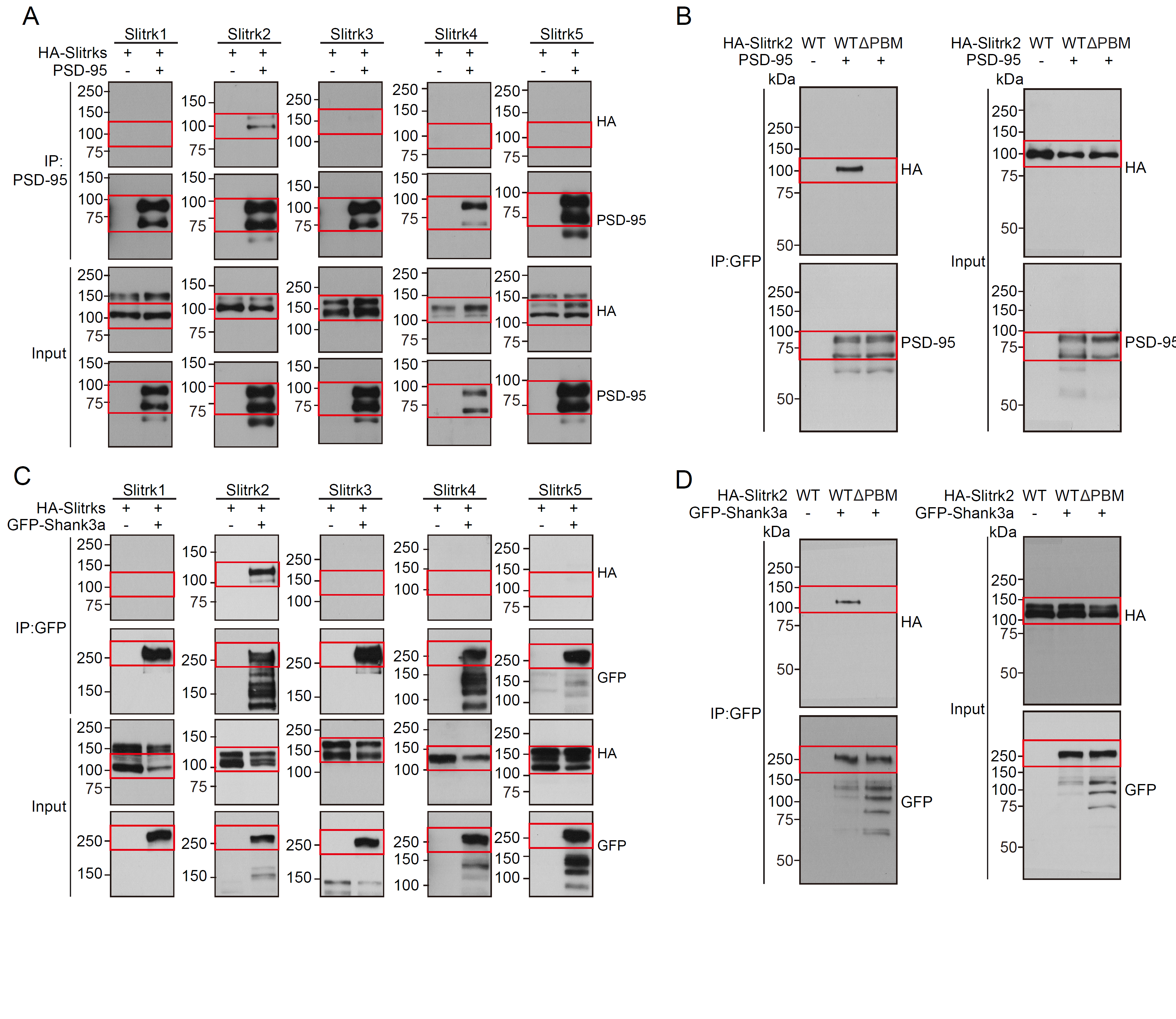


**Supplementary Figure 1.** Uncropped WB images

**(A, B, C)** The original WB images used for Figure 1C(**A**), 1D(**B**), 1F(**C**), and 1G(**D**). Red boxes indicate cropped lines.

**Supplementary Figure 2.** Uncropped WB images

**(A, B)** The original WB images used for Figure 2A(**A**), and 2B(**B**). Asterisks indicate non-specific bands. Red boxes indicate cropped lines.


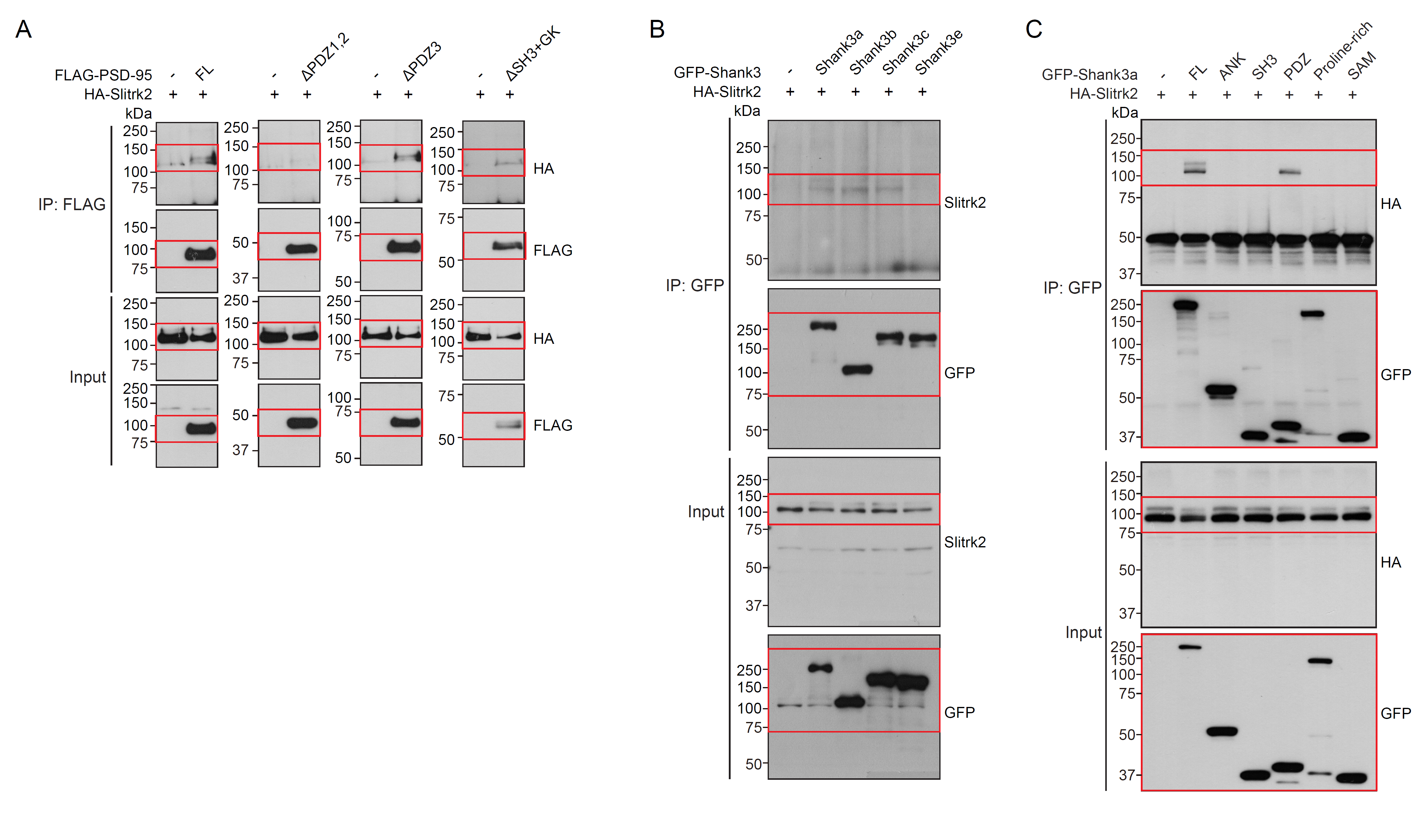


**Supplementary Figure 3.** Uncropped WB images

**(A, B, C)** The original WB images used for Figure 3B(**A**), 3D(**B**), and 3F(**C**). Red boxes indicate cropped lines.


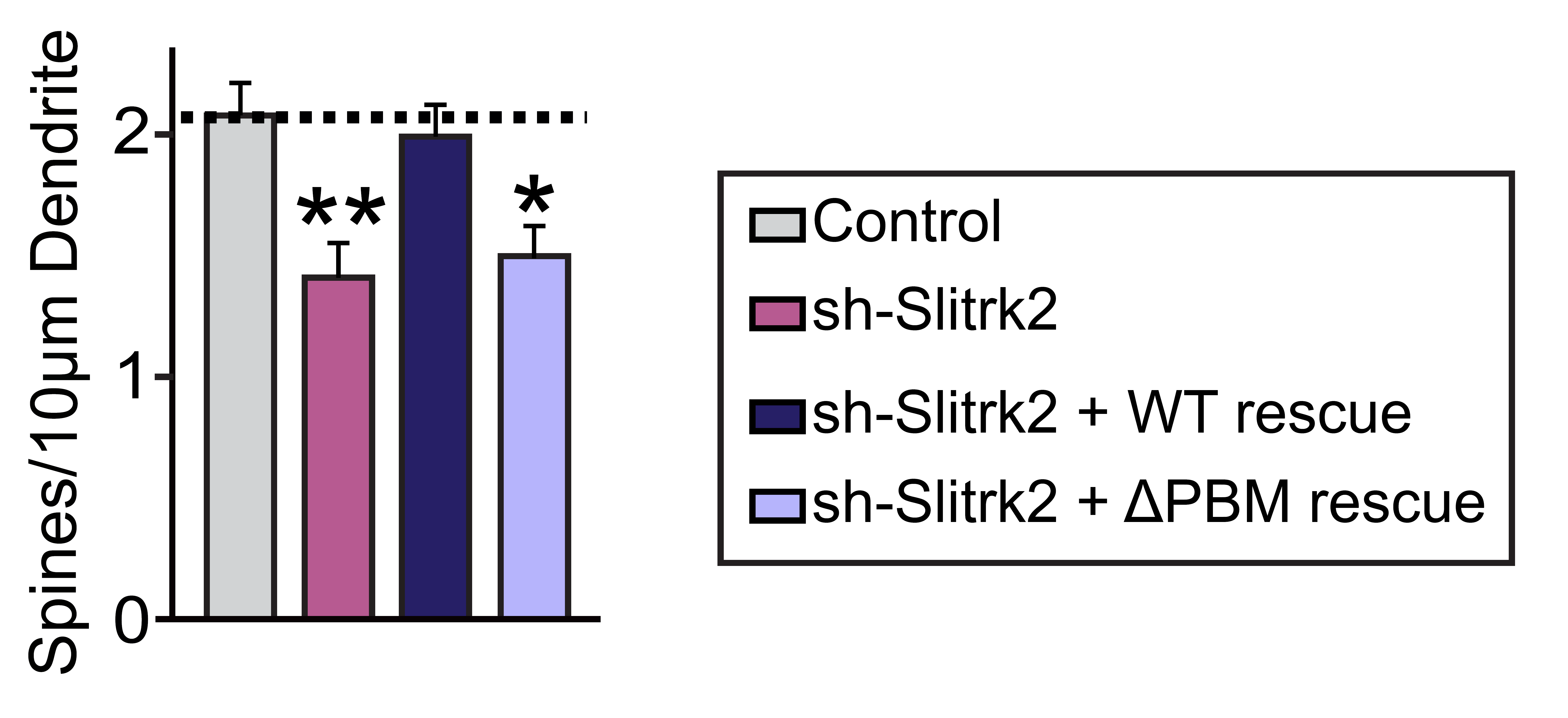


**Supplementary Figure 4.** The effects of Slitrk2 knockdown in neurons on dendritic spine density.

Cultured hippocampal neurons were transfected with a lentiviral vector expressing sh-Control, sh-Slitrk2, or coexpressing sh-Slitrk2 and shRNA-resistant Slitrk2 WT or Slitrk2 ΔPBM at DIV8 and analyzed at DIV14 by immunofluorescence staining for EGFP. Graph indicates the number of dendritic spines. Data are presented as means ± SEMs from three independent experiments (n = 22–30 neurons; **p* < 0.05, ***p* < 0.01 vs. control; non-parametric ANOVA with Kruskal-Wallis test followed by *post hoc* Dunn’s multiple comparison test).

**Supplementary Figure 5.** Analysis of AAV expression in the hippocampal CA1 region.

(**A**) Representative images of the AAV-infected neurons in hippocampal CA1 regions of the mouse brain coronal section (-1.8 mm relative to Bregma), immunostained for EGFP (green), and counterstained with DAPI (blue). Scale bar: 1 mm.

(**B**) Representative images of hippocampal CA1 pyramidal cell layers 2 weeks after stereotactic injection of AAVs expressing sh-Slitrk2 or coexpressing sh-Slitrk2 and HA-tagged Slitrk2 WT (+ Slitrk2 WT) or HA-tagged Slitrk2 ΔPBM (+ Slitrk2 ΔPBM), immunostained for EGFP (green) and Slitrk2 or HA (red), and counterstained with DAPI (blue). Scale bar: 20 μm (applies to all images).

(**C**) Immunoblotting analyses with Slitrk2 antibodies to show the knockdown efficacy of sh-Slitrk2 *in vivo*. Infected mouse brain lysates were collected after stereotactic injection of AAVs expressing sh-Slitrk2 or coexpressing sh-Slitrk2 and HA-tagged shRNA-resistant Slitrk2 WT (+ S2 WT) or HA-tagged Slitrk2 ΔPBM (+ S2 ΔPBM), and immunoblotted with anti-Slitrk2 antibodies. anti-β-actin antibodies were used as normalization controls. An asterisk indicates non-specific bands.
